# A Rare Image of Bladder Herniation Concealed in a Frequent Diagnosis

**DOI:** 10.7759/cureus.31663

**Published:** 2022-11-19

**Authors:** José Miguel Lessa Silva, Joana Gomes Cochicho

**Affiliations:** 1 Internal Medicine, Hospital Dr. José Maria Grande, Portalegre, PRT

**Keywords:** scrotal cystocele, hernia reduction, computerized tomography scan, urinary retention, bladder hernia, urinary tract infection

## Abstract

Herniation of the urinary bladder is a relatively uncommon condition, and when it herniates to the scrotum, it can also be called a scrotal cystocele. The incidence of inguinal herniation is estimated to vary between 1% and 3 % of all inguinal hernias. However, scrotal cystocele is believed to occur in less than 1% of cases and only 7% are diagnosed preoperatively.

We present a case of a 91-year-old male who presented to our hospital with dysuria, fever, right scrotal pain, and tumefaction. In the course of the patient's evaluation, a CT scan of the pelvis was performed, which showed a right inguinal hernia constituting the bladder.

A hernia reduction was performed, and it led to immediate symptomatic relief. After the procedure, antibiotics were initiated and administrated for the urinary tract infection.

Bladder hernia and its extension to the scrotum is rare; therefore, this is a relevant case that may contribute to additional knowledge in the medical community.

## Introduction

A urinary tract infection is one of the most common infections [1], in all ages and sexes. It has variable severity and may manifest as simple cystitis or be as severe as urosepsis.

The use of computerized tomography in the evaluation of these patients is usually reserved for complications or therapeutic failure [2].

On the other hand, herniation of the urinary bladder is an uncommon situation, with an incidence of 1%-3% in men over the age of 50 years [3,4]. The herniation of the scrotum was first described by Levine in 1951 and defined as scrotal cystocele [5]. The incidence of scrotal cystocele is believed to occur in less than 1% of the cases, and only 7% are diagnosed preoperatively [6].

Bladder herniation generally remains asymptomatic and is discovered incidentally. However, symptoms such as dysuria, urgency, nocturia, and hematuria are common [7].

Our case aims to increase awareness about the great utility that medical imaging provides in further diagnosing complicated urinary tract infections and present the medical community with a rare example of scrotal cystocele.

## Case presentation

We present a 91-year-old male, with a documented medical history of arterial hypertension, ischemic cardiopathy, obesity, anemia, duodenal ulcer, and dyslipidemia.

The patient was seen in the emergency department with a chief complaint of dysuria, fever, and right scrotal tumefaction. The patient denied urgency, pollakiuria, or hematuria.

In the physical examination, tumefaction and pain in the hypogastric area were evident, suggestive of bladder globus, associated with edema and intense pain in the right scrotum. A prostate examination was denied by the patient.

The urinary retention prompted urinary catheter introduction, and 2,500 mL of urine was removed.

Laboratory examination showed an increased C-reactive protein (296 mg/L) and leukocytosis (17,400 mm^-3^). In urinalysis, pyuria was evident.

**Figure 1:**
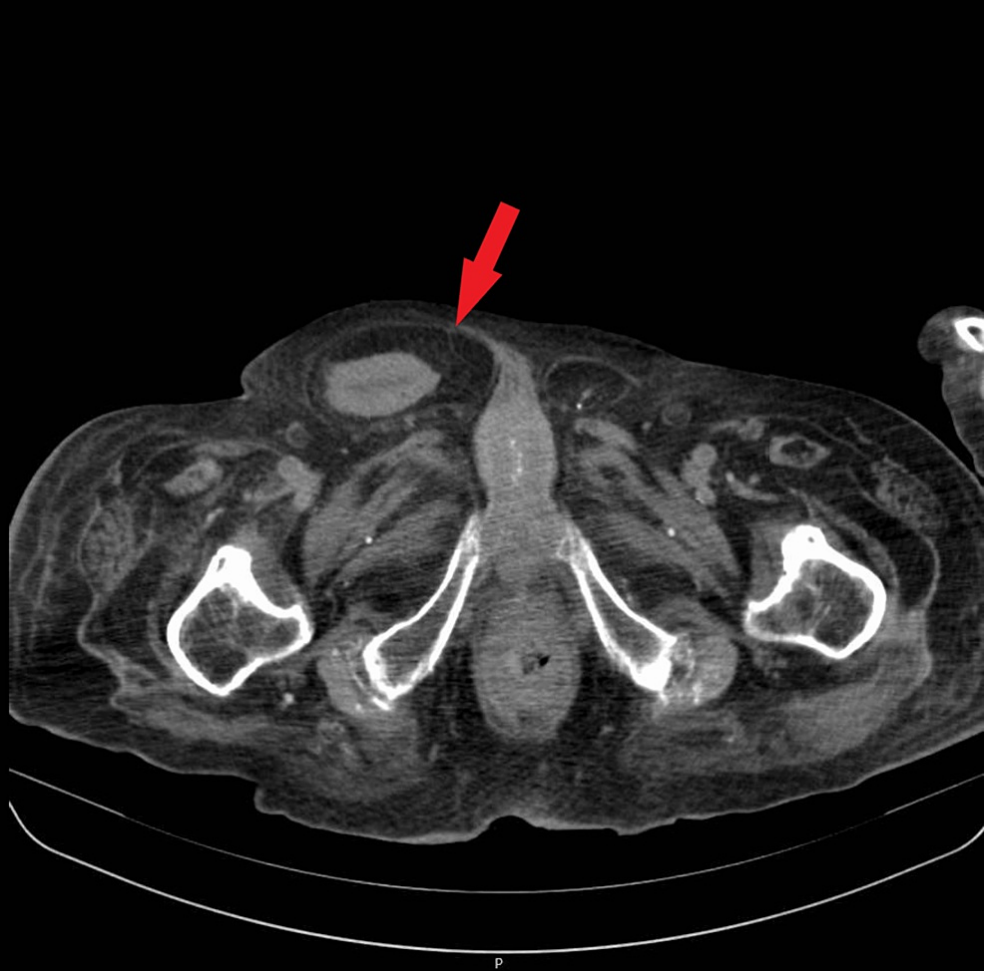
Inguinoscrotal hernia reaching the scrotum. (The bladder can be seen reaching the inferior region of the scrotum.)

**Figure 2:**
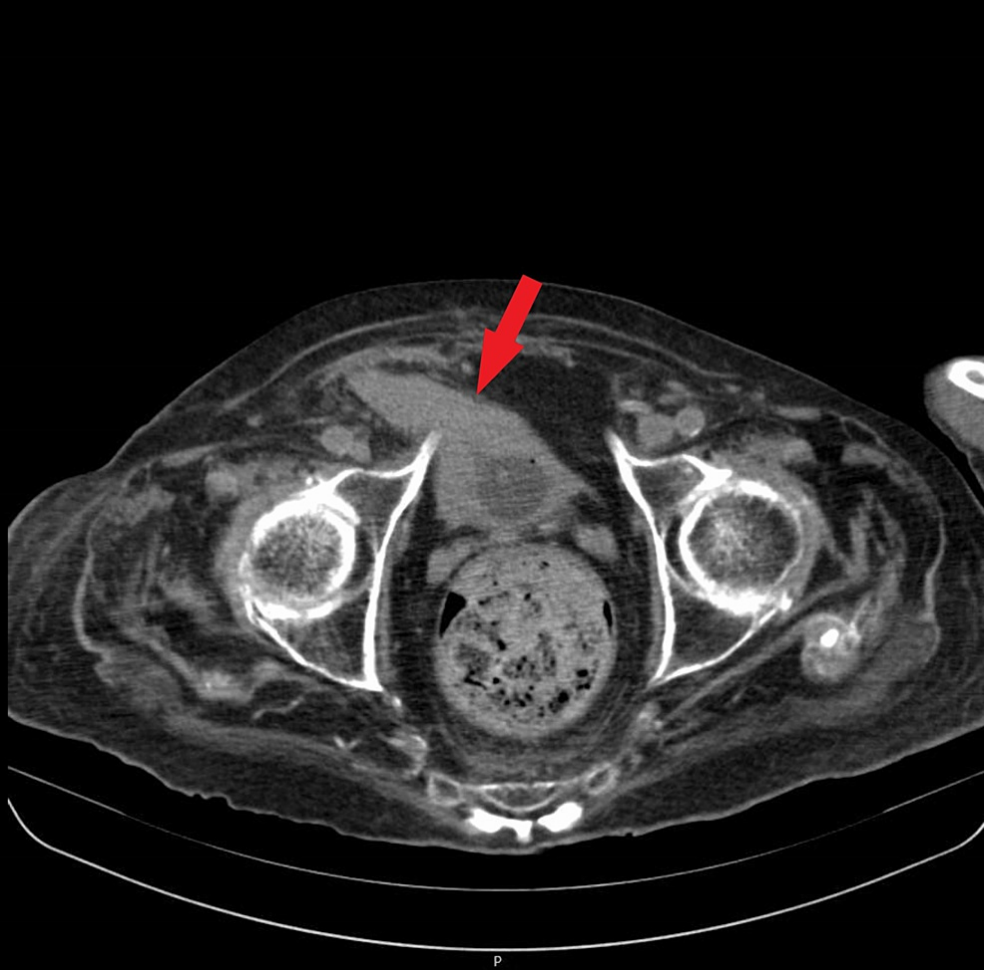
Inguinoscrotal hernia. (Bladder herniation can be seen.)

The inguinal-scrotal symptoms warranted further radiological evaluation. At the time, ultrasonography was not accessible; therefore, a pelvic CT scan was ordered. Contrast was not administered due to the patient history of the previous reaction. The CT scan showed a right inguinal hernia constituting a bladder reaching the inferior region of the scrotum (Figure 1 and Figure 2).

After evaluation by the surgical team, the patient was deemed unfit for surgery. A manual hernia reduction was performed instead, with a taxis maneuver. The hernia reduction led to immediate symptomatic relief. Thereafter, antibiotic therapy with cefuroxime was started according to local guidelines.

## Discussion

Inguinal hernia is a rare clinical entity, which particularly affects obese and elderly patients [7].

Inguinal bladder hernias are oftentimes formed as a result of chronic bladder distention [7]. In the case of our patient, he was retaining a significant amount of urine and had a urinary tract infection. We also considered the possibility that this hernia could have been previously established, related to the patient's characteristics, such as obesity and age. This could have led to urinary stasis, thereby predisposing the patient to infection.

An exuberant indirect inguinal hernia, which was formed by the bladder reaching the inferior region of the scrotum, happens in less than 1% of the cases of inguinal hernias, as shown in Figures 1-2 [6]. We also highlight the singularity of the association between the entities [8].

## Conclusions

It is important to perform a thorough physical examination, and in instances where further evaluation is warranted, one must consider radiological imaging for more information. In this case, a seemingly simple urinary tract infection was associated with a rare anatomical finding of a bladder hernia.
